# *MAO-B* Polymorphism Associated with Progression in a Chinese Parkinson's Disease Cohort but Not in the PPMI Cohort

**DOI:** 10.1155/2022/3481102

**Published:** 2022-09-17

**Authors:** Shi-Shuang Cui, Ling-Yu Wu, Gen Li, Juan-Juan Du, Pei Huang, Jin Liu, Yun Ling, Kang Ren, Zhong-Lue Chen, Sheng-Di Chen

**Affiliations:** ^1^Department of Neurology and Institute of Neurology, Ruijin Hospital, Shanghai Jiao Tong University School of Medicine, Shanghai, China; ^2^Department of Geriatrics, Ruijin Hospital, Shanghai Jiao Tong University School of Medicine, Shanghai, China; ^3^Gyenno Science CO., LTD., Shenzhen, China

## Abstract

**Introduction:**

Genetic factors play an important role in Parkinson's disease (PD) risk. However, the genetic contribution to progression in Chinese PD patients has rarely been studied. This study investigated genetic associations with progression based on 30 PD risk loci common in a longitudinal cohort of Chinese PD patients and the Parkinson's Progression Markers Initiative (PPMI) cohort.

**Methods:**

PD patients from the true world (TW) Chinese PD longitudinal cohort and the PPMI cohort with demographic information and assessment scales were assessed. A panel containing 30 PD risk single nucleotide polymorphisms was tested. Progression rates of each scale were derived from random-effect slope values of mixed-effects regression models. Progression rates of multiple assessments were combined by using principal component analysis (PCA) to derive scores for composite, motor, and nonmotor progression. The association of genetic polymorphism and separate scales or PCA progression was analysed via linear regression.

**Results:**

In the Chinese PD cohort, *MAOB* rs1799836 was associated with progression based on the Montreal Cognitive Assessment, the top 3 principal components (PCs) of nonmotor PCA and PC1 of the composite PCA. In the PPMI cohort, both MDS-Unified Parkinson's Disease Rating Scale II and motor PC1 progression were associated with *RIT2* rs12456492. The *PARK16* haplotype was associated with Geriatric Depression Scale and the State-Trait Anxiety Inventory for Adults progression, and the *SNCA* haplotype was associated with the Hoehn-Yahr staging progression and motor PC1 progression. Ethnicity-stratified analysis showed that the association between *MAOB* rs1799836 and PD progression may be specific to Asian or Chinese patients.

**Conclusion:**

*MAOB* rs1799836 was associated with the progression of nonmotor symptoms, especially cognitive impairment, and the composite progression of motor and nonmotor symptoms within our Chinese PD cohort. The *RIT2* rs12456492 and *SNCA* haplotypes were associated with motor function decline, and the *PARK16* haplotype was associated with progression in mood in the PPMI cohort.

## 1. Introduction

Parkinson's disease (PD) is the second most common neurodegenerative disease in the world and is characterized by motor disabilities, including tremor, bradykinesia, rigidity, and gait disorder, as well as nonmotor symptoms, including hyposmia, REM sleep behaviour disorder (RBD), cognitive impairment, and others. However, the progression of these different clinical features varies among individuals, and the mechanisms underlying this heterogeneity are not well understood.

There are a number of genes that increase the risk for PD in a largely Mendelian fashion, including *SNCA*, *LRRK2*, *VPS35*, *Parkin*, *PINK1*, *DJ1*, and so on [1]. Genome-wide association studies (GWASs) of PD have identified 90 independent loci associated with disease risk, with an overall heritability estimated to be between 22% and 27% [2–4]. Several studies have explored certain gene mutations, such as those in *LRRK2*, *Parkin*, *SNCA*, *GBA*, and others, with distinct clinical presentations and motor or cognitive impairment progression [5–9]. Nevertheless, these studies were either based on cross-sectional PD cohorts or involved limited genetic variants. Although a recent GWAS revealed that *APOE4* was associated with cognitive progression and *ATP8B2* was associated with motor progression in longitudinal PD cohorts [7], a longitudinal genome-wide survival study revealed that *RIMS2, TMEM108, WWOX, GBA,* and *APOE* were involved in cognitive impairment progression in PD patients [10]. However, the genetic contribution to progression in Chinese PD patients has rarely been studied. Thus, the current study investigated genetic associations with progression in a longitudinal PD cohort based on 30 PD risk loci common in Chinese patients. To consider the inconsistent frequency of PD risk loci between different races, we also explored the associations between genetics and PD progression in a Parkinson's Progression Markers Initiative (PPMI) cohort in which the Caucasian population was the majority.

## 2. Methods

### 2.1. Patients and Grouping

The true world longitudinal Chinese PD (TW) cohort included outpatients from November 2014 to September 2017 from the Department of Neurology, Ruijin Hospital, affiliated to Shanghai Jiao Tong University School of Medicine. These patients were enrolled according to the following criteria: (1) a diagnosis of PD according to Movement Disorder Society Clinical Diagnostic Criteria [11]; (2) available clinical assessment scores; (3) multiple visiting records in the Health Information System; and (4) previous genetic testing related to PD. All the diagnoses of PD patients were made by the senior movement disorder specialist. The exclusion criteria were secondary parkinsonism caused by other neurological conditions, such as vascular, inflammatory, toxic, and drug-induced parkinsonism, or parkinsonism plus syndrome including progressive supranuclear palsy, cortico-basal degeneration, and multiple system atrophy. This study was approved by the local ethics committees of Shanghai Ruijin Hospital. Written informed consent was obtained from all TW participants. As indicated in Figure S1, 50 patients were included for further analyses.

For the PPMI cohort, approval was obtained to download and analyze the publicly accessible whole-genome sequence (WGS) and clinical data [12]. Patients whose longitudinal follow-up evaluations were not consistent with a diagnosis of PD were excluded.

### 2.2. Clinical Assessments

The collection of demographic and clinical data included age, sex, education, and family history of PD. Overlapping scales shared by the two cohorts were collected to perform genotype-phenotype association analyses (Table S1); these scales included Hoehn-Yahr (H-Y) staging, MDS-Unified Parkinson's Disease Rating Scale (MDS-UPDRS), Montreal Cognitive Assessment (MoCA), Scales for Outcomes in Parkinson's Disease—Autonomic Dysfunction (SCOPA-AUT), Epworth Sleepiness Scale Score (ESS), Hamilton Anxiety Rating Scale (HAMA) in the TW cohort or State-Trait Anxiety Inventory for Adults (STAI) in the PPMI cohort, Rapid Eye Movement Sleep Behavior Disorder Hong Kong (RBD-HK) in the TW cohort or Rapid Eye Movement Sleep Behavior Disorder Questionnaire (RBD-Q) in the PPMI cohort, and Hamilton Rating Scale for Depression (HAMD)-17 in the TW cohort or Geriatric Depression Scale (GDS) in the PPMI cohort. In the PPMI cohort, we used motor assessments conducted in the “off” medication state.

### 2.3. Genetic Testing and Haplotype Block Construction

A panel containing 30 PD-related single nucleotide polymorphisms (SNPs) was designed and tested in the TW PD patients. The genotyping methods were previously reported [13]. Peripheral blood samples were collected, and DNA was extracted from leukocytes by using the phenol–chloroform isopropyl alcohol method. Primers were designed by using Primer 5 (version 5.00, PREMIER Biosoft International). After purification of polymerase chain reaction products by both phosphorylase (FastAP, Applied Biosystems) and exonuclease I (EXO I, Applied Biosystems), the extension reaction was performed by using the SNaPshot Multiplex kit from ABI. The extended products were further purified by using phosphorylase (FastAP, Applied Biosystems) and sampled by using ABI3730xl (Applied Biosystems). The results of SNP typing were analyzed by using Genemap 4.0 (Applied Biosystems). Population structure was determined by using EIGENSOFT v6.1.4 [14]. We excluded rs2230288 from our analysis because all the patients in the TW cohort had the G/G genotype at this locus.

For the PPMI cohort, variants of interest were extracted from whole-genome sequencing (WGS) data (project 118, hg38 aligned January 2021 VCFs) using BCFtools [15].

For the construction of haplotype blocks, two steps were followed: (1) a list of variants was pruned based on linkage disequilibrium (LD) among 29 SNPs in the CHB, CHS, and CEU populations of 1 KG, and those pruned variants on the same chromosome were grouped as LD blocks in the PPMI and TW cohorts (Figure S2), and (2) haplotype alleles were constructed through PHASE version 2.1 [16, 17]. Considering previous studies reporting LD of *LRRK2*, the *LRRK2* haplotype was also assigned in the analysis in addition to the two SNPs [6]. Thus, a total of 3 LD blocks were created, namely, the *PARK16* haplotype, *SNCA* haplotype, and *LRRK2* haplotype blocks, and the frequencies of the haplotype alleles are listed in Table S2. Finally, 21 SNPs and 3 LD blocks were included to perform the genotype-phenotype association analyses (Figure S3). All the genetic information collected from the PPMI Cohort Genetic Database (*N* = 1830) and the TW cohort (*N* = 415) was used to construct haplotypes (Figure S1). The LDlinkR package in R was employed to construct the haplotypes.

### 2.4. Progression and PCA Progression

Progression rates were derived from random-effect slope values of mixed-effects regression models for two populations [18]. The disease status of PD patients is always assessed by multiple clinical scales, and significant correlations existed between scales in the TW cohort (Table S3). Thus, the collinearity between these scales implied redundancy and made it difficult to clarify genotype-phenotype associations. To further investigate the specific influence of gene loci on the progression of motor and nonmotor PD symptoms, we performed principal component analysis (PCA) on the motor or nonmotor symptom progression rate after zero centring and scaling to have unit variance after a combination of TW and PPMI PD populations [18]. We found that the first principal components (PCs) of motor and nonmotor progression were moderately correlated (*r* = −0.435, *P* < 2.2 × 10 − 16). Therefore, the third PCA was conducted to combine all the motor and nonmotor measures to create a composite progression score to illustrate the composite progression of PD. PC1 from this cross-domain PCA accounted for 37.61% of the joint variance. The correlations of polymorphisms versus nonmotor PC1-PC3, motor PC1-PC2, and composite PC1 were considered (Table S4), and the contributions of variables to the PCs are illustrated in Table S5 and Figure S4.

### 2.5. Ethnicity-Stratified Genotype-Phenotype Correlation

The distinct genotype-phenotype correlation might be caused by population race since the genetic background varied between the PPMI and Chinese PD cohorts mentioned above. To illustrate whether there were ethnicity-specific associations, we employed an ethnicity-stratified association analysis. Since the PPMI PD cohort is a multi-ethnic cohort, among which there were 346 Caucasian people (346/377, 92%), 7 Asian people (7/377, 1.8%), 6 black people, and 18 individuals of other races, the TW cohort and the PPMI cohort were merged and then secondarily grouped into four other cohorts: the PPMI Caucasian, PPMI Asian, TW, and All Asian (merging the PPMI Asian and TW cohorts) cohorts. Genotype-phenotype associations were compared across those 4 groups. Considering the limited sample size of the PPMI Asian cohort, both mild (*P* < 0.05) and strict (*P* < 0.002381) association settings were used to explore differences and commonalities with pooled raw progression and PCA progression. Those polymorphisms were defined as mono-ethnic specific genetic features, which were merely correlated with raw progression or PCA progression in mono-ethnic patients.

### 2.6. Statistical Analysis

R (version 4.1.0) with R Studio (version 1.4.1717) was used to perform the statistical analysis. The Factoextra package was used to extract and visualize the PCA results, and other visualization was performed by using ggplot2. A linear mixed model was constructed by using the lme4 package.

All continuous data are presented as the median (IQR) and were compared by using the Mann‒Whitney test. Categorical data are presented as *N* (%) and were compared by using the chi-square test. A *P* value < 0.05 (two-tailed) was considered statistically significant. The association between the SNPs or haplotypes and the progression of clinical assessments was performed using a linear regression model and partial correlation with adjustment for sex, age onset, education, and disease duration at baseline under an additive minor genetic model. Bonferroni correction was used for multiple comparisons. A *P* value < 0.002381 (two-tailed) was considered statistically significant after Bonferroni correction.

## 3. Results

Table 1 presents the demographic information of the TW PD and PPMI PD cohorts. The TW cohort included 50 PD patients with an average follow-up of 1.33 years, and the PPMI cohort included 413 PD patients with an average follow-up of 4.35 years. The TW and PPMI PD cohorts were comparable regarding disease duration (Table 1), while the TW patients were significantly younger and had a lower education level than the PPMI patients (Table 1). Despite 30 common variants, genetic background discrepancies were detected in the two cohorts (Figure S5), and the genotype frequency of most variants was significantly different between the two cohorts (Table S6). PCA progression rates were comparable in the two cohorts; thus, we combined the two cohorts to perform PCA.

### 3.1. Genotype-Phenotype Association in the TW Cohort

A linear mixed-effect model was used to calculate patient-specific disease progression in the two cohorts separately. An adjusted linear regression model with additive genetic assumption was used to assess the association between disease progression rates and gene polymorphisms (21 SNPs and 3 LD blocks). Age at baseline, sex, disease duration in years, and education level were selected as covariates. In the TW cohort, progression measured by the MoCA total score was significantly associated with B-type monoamine oxidation (*MAO-B*) rs1799836. One additional C allele led to 0.967 (0.495–1.439) more of the corresponding progression rate (P=0.0001878) (Table 2). LD SNPs were grouped into LD blocks (noted *PARK16* haplotype, *SNCA* haplotype, and *LRRK2* haplotype). No multiple-testing significant correlation between LD blocks and progression of separated scales was found.

For PCA-based progression rates, we found that *MAO-B* rs1799836 was associated with PC1 (*P*=0.0000765), PC2 (*P*=0.0000686), and PC3 (*P*=0.0000084) of nonmotor progression PCA and PC1 (*P* < 0.0001) of composite progression PCA (Table 3). No multiple-testing significant or mild correlation was detected between LD blocks and PCs of the three types of PCA.

### 3.2. Genotype-Phenotype Association in the PPMI Cohort

In the PPMI cohort, both progressions measured by MDS-UPDRSII (*P*=0.0021562) and PC1 of motor progression PCA (*P*=0.0013333) were associated with *SYT4.RIT2* rs12456492, and one more copy of the *G* allele led to 0.286 (0.104–0.468) and −0.333 (−0.536−0.131) increases in the corresponding progression rate, respectively (Tables 2 and 3). The *PARK16* haplotype was associated with progression measured by GDS scores and STAI scores, while the *SNCA* haplotype was associated with progression based on H-Y staging and PC1 of motor progression PCA (Table 4).

### 3.3. Ethnicity-Stratified Genotype-Phenotype Association

**Figure 1:**
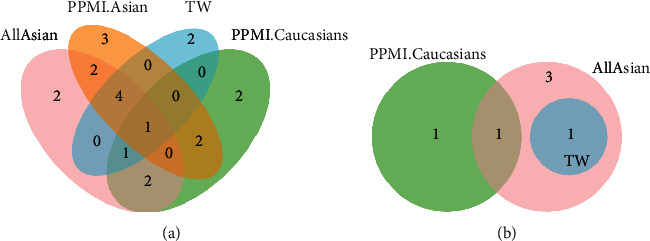
Venn plot of ethnicity-stratified correlation analysis. (a) Venn plot of ethnicity-stratified correlation analysis with *P* < 0.05. (b) Venn plot of ethnicity-stratified correlation analysis with *P* < 0.002381.

The significant ethnicity-stratified genotype-phenotype association (*P* < 0.05) is listed in Table S6. As indicated in Table 5 and Figure 1, we obtained 4 Asian- and 1 Caucasian-specific strict-progression-correlated polymorphisms (*PARK16* haplotype, *MAO-B, NUCKS1*, and *PRKN*; *SYT4.RIT2*), and 1 shared polymorphism among Asians and Caucasians (*SNCA* haplotype) based on strict setting association (*P* < 0.002381).

Since most Asians were from the TW cohort and all Caucasians were from the PPMI cohort, ethnicity-specific polymorphisms are likely biased by cohort. Thus, we investigated the genotype-phenotype association within the PPMI Asian cohort with a sample size of 7 PD patients. Considering the limited sample size, we set the statistical significance level *a*=0.05 and found 10 unique genetic polymorphisms in the PPMI Asian cohort. Among them, the association between *MAO-B* and MoCA again was significant, as was the association between *SYT4.RIT2* and H-Y staging (Table 5). In addition, when all the associations in the PPMI Asian cohort were sorted by the negative logarithm of the P value, the former was the 6th significantly associated gene, while the latter was the 11th (Table S7).

## 4. Discussion

In our study, we explored the association of 30 PD risk loci common in Chinese patients with PD progression assessed by comprehensive scales in a TW PD cohort, which has rarely been reported in previous studies. In our Chinese cohort, we found that *MAO-B* rs1799836 was associated with PD progression, which may be specific to Asian or Chinese populations. Furthermore, we also performed the analysis of the association between haplotype and PD progression, as most previous studies performed the analysis based on SNPs. In addition to using the traditional method of analyzing the association of SNPs and raw scale progression in PD separately, a new method of analyzing clinical progression in PD was used in our clinical cohorts by combining multiple assessments in a data-driven PCA to derive scores of composite, motor, and nonmotor progression, combining multiple measures to identify latent components that explain the most variability in the data and reflect disease progression more accurately.

Two main findings were observed in our Chinese cohort. First, we found that *MAO-B* rs1799836 was associated with a change in MoCA scores in our TW cohort. Previous studies found that *MAO-B* rs1799836 was associated with the risk of PD in Chinese and Asian populations, whereas this association was insignificant in the Caucasian population [19–21]. A randomized, double-blind, placebo-controlled prospective study found that rasagiline, a selective monoamine oxidase type-B inhibitor, exerts beneficial effects on attention and executive functions in nondemented but cognitively impaired PD patients [22]. Another study found that A*β*42 levels were also decreased in carriers of the A allele in the *MAO-B* rs1799836 polymorphism [23]. These studies indicated that the *MAO-B* gene may be associated with cognition in PD patients. To further improve the phenotypic measure, we used the PCA data reduction method and found that *MAO-B* rs1799836 was associated with PC1-3 within nonmotor progression PCA and PC1 of composite progression PCA, indicating that *MAO-B* rs1799836 may be associated with nonmotor progression and composite progression of PD. One possible mechanism is that MAO-B plays a role in degrading norepinephrine (NE) in addition to dopamine (DA), as NE involves many processes, such as cognitive functions, sleep, pain modulation, mood, autonomic functions, blood‒brain barrier permeability, neuroprotection, and immunological mechanisms in the brain, all of which have been impaired in PD and related to nonmotor symptoms [24, 25].

The finding that *MAO-B* rs1799836 was associated with PD progression in our Chinese cohort was seldom or never reported in previous studies. As the cohorts used in the previous studies included very few Asian patients, *MAO-B* rs1799836 might be the Chinese- or Asian-specific SNP associated with PD progression. Thus, we explored the associations of the 30 PD risk loci common in Chinese patients and PD progression in the PPIM cohort, which included mostly Caucasian patients, and found that *RIT2* rs12456492 was associated with the change in MDS-UPDRS II and dim1 of motor progression PCA, while no significant association was found between *MAO-B* rs1799836 and PD progression. The protein encoded by the human *RIT2* gene binds to the product of human calmodulin 1 (CALM1), and the latter binds to *α*-synuclein and microtubule-associated protein tau, suggesting that *RIT2* may share the same pathway as *SNCA* and *MAPT* in the pathogenesis of PD [26–28]. The association of *RIT2* rs12456492 with PD risk among the Caucasian population has been investigated and found to be significant [29]. The relevant results in Asian populations in different studies have been inconsistent, but a recent meta-analysis of Asian populations demonstrated that *RIT2* rs12456492 may increase PD risk in Asian individuals [30], while *RIT2* rs12456492 was not associated with disease progression in our Chinese PD cohort. In a previous GWAS meta-analysis, a suggestive association of PD motor subtype with *RIT2* rs12456492 was detected, implicating disease progression. In addition to single SNPs, the association of the *SNCA* haplotype with a change in H-Y staging was found in the PPMI cohort. *SNCA* codes for the protein *α*-synuclein, the most important pathologic protein in PD, and is frequently reported to be associated with alpha-synuclein levels and motor function decline [5, 31–33]. *PARK16* polymorphisms, including *NUCKS1* and *RAB29*, are associated with decreased PD risk in previous Asian and Caucasian studies [34]. However, the association of the *PARK16* haplotype and progression in PD has rarely been reported in previous studies.

The inconsistency between our TW cohort and the PPMI cohort may be caused by the different patient ethnicities. Thus, we performed a study within the PPMI Asian population and found that *MAO-B* rs1799836 was significantly associated with cognitive decline at a level of *P* < 0.05. However, we failed to find significance after correction, as only 7 patients were included in this analysis. We also assessed only the Caucasian population of the PPMI cohort and found that *MAO-B* rs1799836 was not associated with PD progression even at a level of *P* < 0.05. In addition, we performed the same analysis within all Asian patients in the 2 cohorts and found that *MAO-B* rs1799836 was associated with PD progression, while RIT2 rs12456492 was not after Bonferroni correction. Thus, we suspected that the association between *MAO-B* rs1799836 and PD progression may be specific in Asian or Chinese populations. However, this analysis was underpowered due to the small sample size, especially in Asia. Thus, a longitudinal study with a large sample size of Asian or Chinese PD patients is needed.

Our study has several limitations. The sample size of our TW study was small. Second, follow-up was limited. The limited follow-up and small sample size may lead to underpower for variants with smaller effects on progression. A third limitation is that we only detected 30 SNPs rather than GWAS. Thus, a larger and longer study of Chinese PD patients is needed.

## 5. Conclusion

*MAO-B* rs1799836 was associated with PD progression in nonmotor symptom, particularly cognitive impairment, and composite progression of motor and nonmotor symptoms in a Chinese PD cohort. The *RIT2* rs12456492 and *SNCA* haplotypes were associated with motor function decline, and the *PARK16* haplotype was associated with progression in mood in the PPMI cohort. The association between *MAO-B* rs1799836 and PD progression may be specific to Asian or Chinese patients.

## Figures and Tables

**Table 1:** Cohort demographics at baseline.

Item	TW (*N* = 50)	PPMI (*N* = 413)
	Median (IQR)/*n* (%)	Median (IQR)/*n* (%)
Age of onset, yrs	58 (9)	60.45 (13.8)
Age, yrs	63 (8.75)	62.37 (13.99)
Duration, yrs	4 (4)	4.23 (5.28)
Educ: 0–6	3 (6.38)	2 (0.48)
Educ: 7–9	17 (36.17)	7 (1.69)
Educ: 10–12	13 (27.66)	65 (15.66)
Educ:≥13	14 (29.79)	341 (82.17)
Sex: male	30 (60)	270 (65.06)
Sex: female	20 (40)	145 (34.94)
Hoehn-Yahr staging	1.5 (1)	2 (1)
MDS-UPDRS	27.5 (30)	31 (17)
MDS-UPDRSI	4 (8)	5 (4)
MDS-UPDRSII	7 (7)	5 (5)
MDS-UPDRSIII	14 (16.75)	20 (12)
MDS-UPDRSIV	0 (0)	—
ESS	4.5 (5)	5 (5)
HAMA or STAI	6 (7)	62 (24)
HAMD-17 or GDS	5 (5)	2 (2)
MoCA	25 (5)	27 (3)
RBD-HK or RBD-Q	7 (23)	3 (3.25)
SCOPA-AUT	7.5 (12)	8 (7)

ESS, Epworth sleepiness scale score; GDS, geriatric depression scale; HAMA, Hamilton anxiety rating scale; HAMD-17, Hamilton rating scale for depression; MDS-UPDRS, MDS-unified Parkinson's disease rating scale; MoCA, Montreal cognitive assessment; PPMI, parkinson's progression markers initiative; RBD-HK, rapid eye movement sleep behavior disorder Hong Kong; RBD-Q, rapid eye movement sleep behavior disorder questionnaire; SCOPA-AUT, scales for outcomes in parkinson's disease-autonomic dysfunction; STAI, state-trait anxiety inventory for adults.

**Table 2:** Association between SNPs and progression of the separate scale in the TW and PPMI cohorts.

Cohort	Gene	SNP	Scale	Estimate (95% CI)	*P* value	REF/ALT
PPMI	*SYT4.RIT2*	rs12456492	MDS-UPDRSII	0.286(0.104–0.468)	0.0021562	A/G
TW	*MAO-B*	rs1799836	MoCA	−0.967(−1.439–0.495)	0.0001878	T/C

MDS-UPDRS, MDS-unified Parkinson's disease rating scale; MoCA, Montreal cognitive assessment.

**Table 3:** Association between SNPs and PCA progression in the TW and PPMI cohorts.

Cohort	Gene	SNP	PC	Estimate (95% CI)	*P* value	Type
PPMI	*SYT4.RIT2*	rs12456492	PC1	−0.333 (−0.536–0.131)	0.0013333	Motor PCA
TW	*MAO-B*	rs1799836	PC2	0.402 (0.219–0.586)	0.0000686	Nonmotor PCA
	*MAO-B*	rs1799836	PC3	1.584 (0.957–2.21)	0.0000084	
	*MAO-B*	rs1799836	PC1	0.457 (0.247–0.666)	0.0000765	
TW	*MAO-B*	rs1799836	PC1	-−839 (−1.336–0.342)	0.0014921	Composite PCA

PC, principal component; PCA, principal component analysis.

**Table 4:** Association between haplotypes and progression in the TW and PPMI cohorts.

Cohort	HaploBlock	Haplotype	Prog	Estimate (95% CI)	*P* value
PPMI	*PARK16* haplotype	ACAG	GDS	6375 (3028–9721)	0.0002068
		ACCA	GDS	6375 (3028–9721)	0.0002068
		ATCA	GDS	6375 (3028–9721)	0.0002068
		GCCA	GDS	6375 (3028–9721)	0.0002068
		GCAG	GDS	6375 (3028–9721)	0.0002069
		GTCA	GDS	6719 (3192–10247)	0.0002070
		ATCG	GDS	6375 (3028–9721)	0.0002068
		ACCG	GDS	430472 (204429–656514)	0.0002077
		ACAG	STAI	42709 (17981–67436)	0.0007535
		ACCA	STAI	42709 (17982–67437)	0.0007534
		ATCA	STAI	42709 (17982–67436)	0.0007534
		GCCA	STAI	42708 (17981–67435)	0.0007534
		GCAG	STAI	42711 (17983–67438)	0.0007531
		GTCA	STAI	45020 (18954–71085)	0.0007534
		ATCG	STAI	42709 (17982–67436)	0.0007534
		ACCG	STAI	2884040 (1213734–4554345)	0.0007563
	*SNCA* haplotype	AGTGCA	Hoehn-Yahr staging	255 (161–348)	<0.000001
PPMI	*SNCA* haplotype	AGTGCA	Motor PC1	−3090 (-4978–1201)	0.0014101

GDS, geriatric depression scale; PC, principal component; STAI, state-trait anxiety inventory for adults.

**Table 5:** Summary of polymorphisms correlated with PD progression among diverse populations.

Correlation setting	Cohort	Polymorphism
Mild setting	PPMI Caucasians	*SYT4.RIT2, PTHLH, PRKN, MAPT, LOC105377329*, *SNCA* haplotype, *PARK16* haplotype, *CCDC62*
	All Asian	*SYT4.RIT2, RAB29, PRKN, PLA2G6, NUCKS1, MAO-B, LRRK2* haplotype*, LRRK2, HLA-DRA, COMT, SNCA haplotype, PARK16* haplotype
	PPMI Asian	*SYT4.RIT2, RAB29, PLA2G6, PARK7, MAPT, MAO-B, LRRK2* haplotype*, LRRK2, LOC105377329, GPNMB, COMT, BST1*
	TW	*SYT4.RIT2, PRKN, PLA2G6, MAO-B, LRRK2* haplotype*, LRRK2 R1628P.H, LRRK2 G2385 R, COMT*
Strict setting	PPMI Caucasians	*SYT4.RIT2, SNCA* haplotype
	All Asian	*PRKN, NUCKS1, MAO-B, SNCA* haplotype*, PARK16* haplotype
	TW	*MAO-B*

## Data Availability

The data used to support the findings of this study are available from the corresponding author upon request.

## Abbreviations

ESS: Epworth sleepiness scale score
GDS: Geriatric depression scale
GWAS: Genome-wide association study
HAMA: Hamilton anxiety rating scale
HAMD-17: Hamilton rating scale for depression
LD: Linkage disequilibrium
MDS-UPDRS: MDS-unified parkinson's disease rating scale
MoCA: Montreal cognitive assessment
PC: Principal component
PCA: Principal component analysis
PD: Parkinson's disease
PPMI: Parkinson's progression markers initiative
RBD: REM sleep behaviour disorder
RBD-HK: Rapid eye movement sleep behavior disorder Hong Kong
RBD-Q: Rapid eye movement sleep behavior disorder questionnaire
SCOPA-AUT: Scales for outcomes in Parkinson's disease—autonomic dysfunction
SNP: Single nucleotide polymorphism
STAI: State-trait anxiety inventory for adults.

## Supplementary Materials

Supplementary Materials
*Table S1.* Clinical assessments. *Table S2*. Constructed haplotype with PHASE. *Table S3.* Correlation between scale-wise progression. *Table S4.* Correlation between raw PD progression and PCA. *Table S5.* PC variance. *Table S6.* Genotype distribution of 30 SNPs. *Table S7*. Diversity of gene-PD-progression correlation among diverse populations. *Figure S1.* Flowchart of the study. *Figure S2*. Chrome-wise pairwise LD. A: PARK16 haplotype; B: SNCA haplotype; C: chr6snps; D: chr12snps. *Figure S3.* Flowchart of SNP preprocessing. *Figure S4*. Heatmap of the contribution of raw scale progressions to PCs derived from three types of PCA. A: contribution of motor scales of PC from motor PCA; B: contribution of nonmotor scales to PC from nonmotor PCA; C: contribution of all scales to PC from composite PCA. Figure S5. PCA of progression rate and 30 SNPs. A. PCA of progression rate; B. PCA of 30 SNPs.

## References

[1] Domingo A., Klein C. (2018). Genetics of parkinson disease. *Handbook of Clinical Neurology*.

[2] Nalls M. A. B. C., Vallerga C. L., Heilbron K. (2019). Identication of novel risk loci, causal insights, and heritable risk for parkinson’s disease: a meta-analysis of genome-wide association studies. *Lancet Neurology*.

[3] Chang D. N. M. A., Nalls M. A., Hallgrímsdóttir I. B. (2017). A meta-analysis of genome-wide association studies identifies 17 new parkinson’s disease risk loci. *Nature Genetics*.

[4] Li G., Cui S., Du J. (2018). Association of GALC, ZNF184, IL1R2 and ELOVL7 with parkinson’s disease in southern Chinese. *Frontiers in Aging Neuroscience*.

[5] Wang G., Huang Y., Chen W. (2016). Variants in the SNCA gene associate with motor progression while variants in the MAPT gene associate with the severity of parkinson’s disease. *Parkinsonism & Related Disorders*.

[6] Oosterveld L. P. A. J. J., Allen J. C., Ng E. Y. (2015). Greater motor progression in patients with parkinson disease who carry LRRK2 risk variants. *Neurology*.

[7] Sun Y. M., Yu H. L., Zhou X. Y. (2021). Disease progression in patients with parkin-related parkinson’s disease in a longitudinal cohort. *Movement Disorders*.

[8] Cui S. S., Fu R., Du J. J. (2021). Sex effects on clinical features in LRRK2 G2385R carriers and non-carriers in parkinson’s disease. *BMC Neuroscience*.

[9] Huang J., Cheng Y., Li C., Shang H. (2022). Genetic heterogeneity on sleep disorders in parkinson’s disease: a systematic review and meta-analysis. *Translational Neurodegeneration*.

[10] Liu G., Peng J., Liao Z. (2021). Genome-wide survival study identifies a novel synaptic locus and polygenic score for cognitive progression in parkinson’s disease. *Nature Genetics*.

[11] Postuma R. B., Berg D., Stern M. (2015). MDS clinical diagnostic criteria for parkinson’s disease. *Movement Disorders*.

[12] Parkinson Progression Marker I. (2011). The parkinson progression marker initiative (PPMI). *Progress in Neurobiology*.

[13] Liu J., Li G., He Y. (2020). The association analysis of GPNMB rs156429 with clinical manifestations in Chinese population with parkinson’s disease. *Frontiers in Genetics*.

[14] Patterson N., Price A. L., Reich D. (2006). Population structure and eigenanalysis. *PLoS Genetics*.

[15] Danecek P., Bonfield J. K., Liddle J. (2021). Twelve years of SAMtools and BCFtools. *GigaScience*.

[16] Stephens M. S. N., Smith N. J., Donnelly P. (2001). A new statistical method for haplotype reconstruction from population data. *American Journal of Human Genetics*.

[17] Stephens M. D. P., Donnelly P. (2003). A comparison of bayesian methods for haplotype reconstruction from population genotype data. *American Journal of Human Genetics*.

[18] Tan M. M. X., Lawton M. A., Jabbari E. (2021). Genome-wide association studies of cognitive and motor progression in parkinson’s disease. *Movement Disorders*.

[19] Kiyohara C., Miyake Y., Koyanagi M. (2011). Genetic polymorphisms involved in dopaminergic neurotransmission and risk for parkinson’s disease in a Japanese population. *BMC Neurology*.

[20] Liu J. J., Wang W., Meng M., Liang C. S., Zhang J. (2016). Association between monoamine oxidase B A644G polymorphism and parkinson’s disease risk: a meta-analysis in the Chinese population. *Genetics and Molecular Research*.

[21] Sun Y. X., Wang X. H., Xu A. H., Zhao J. H. (2014). Functional polymorphisms of the MAO gene with parkinson disease susceptibility: a meta-analysis. *Journal of the Neurological Sciences*.

[22] Hanagasi H. A., Gurvit H., Unsalan P. (2011). The effects of rasagiline on cognitive deficits in parkinson’s disease patients without dementia: a randomized, double-blind, placebo-controlled, multicenter study. *Movement Disorders*.

[23] Babic Leko M., Nikolac Perkovic M., Klepac N. (2020). Relationships of cerebrospinal fluid alzheimer’s disease biomarkers and COMT, DBH, and MAOB single nucleotide polymorphisms. *Journal of Alzheimer’s Disease*.

[24] Betts M. J., Kirilina E., Otaduy M. C. G. (2019). Locus coeruleus imaging as a biomarker for noradrenergic dysfunction in neurodegenerative diseases. *Brain*.

[25] Dorszewska J. P. M., Prendecki M., Oczkowska A., Rozycka A., Lianeri M., Kozubski W. (2014). Polymorphism of the COMT, MAO, DAT, NET and 5-HTT genes, and biogenic amines in parkinson’s disease. *Current Genomics*.

[26] Lee C. H. J., Della N. G., Chew C. E., Zack D. J. (1996). Rin, a neuron-specific and calmodulin-binding small Gprotein, and rit define a novel subfamily of ras proteins. *Journal of Neuroscience*.

[27] Lee D. L. S., Lee S. Y., Lee E. N., Chang C. S., Paik S. R. (2002). alpha-Synuclein exhibits competitive interaction between calmodulin and synthetic membranes. *Journal of Neurochemistry*.

[28] Padilla R. M. R., Maccioni R. B., Avila J. (1990). Calmodulin binds to a tubulin binding site of the microtubuleassociated protein tau. *Molecular and Cellular Biochemistry*.

[29] Pankratz N., Beecham G. W., DeStefano A. L. (2012). Meta-analysis of parkinson’s disease: identification of a novel locus, RIT2. *Annals of Neurology*.

[30] Liu T. W., Wu Y. R., Chen Y. C., Fung H. C., Chen C. M. (2020). Association of RIT2 and RAB7L1 with parkinson’s disease: a case-control study in a Taiwanese cohort and a meta-analysis in Asian populations. *Neurobiology of Aging*.

[31] Wu-Chou Y. H., Chen Y. T., Yeh T. H. (2013). Genetic variants of SNCA and LRRK2 genes are associated with sporadic PD susceptibility: a replication study in a Taiwanese cohort. *Parkinsonism & Related Disorders*.

[32] Pedersen C. C., Lange J., Forland M. G. G., Macleod A. D., Alves G., Maple-Grodem J. (2021). A systematic review of associations between common SNCA variants and clinical heterogeneity in parkinson’s disease. *NPJ Parkinsons Dis*.

[33] Ritz B., Rhodes S. L., Bordelon Y., Bronstein J. (2012). *α*-Synuclein genetic variants predict faster motor symptom progression in idiopathic parkinson disease. *PLoS One*.

[34] Peeraully T., Tan E. K. (2012). Genetic variants in sporadic parkinson’s disease: east vs west. *Parkinsonism & Related Disorders*.

