# Supplementary material for: MAO-B Polymorphism Associated with Progression in a Chinese Parkinson's Disease Cohort but Not in the PPMI Cohort
Source: Parkinsons Dis. 2022 Sep 17;2022:3481102. doi: 10.1155/2022/3481102 (PMC9509281; doi:10.1155/2022/3481102)
Supplement: Supplementary Materials — Table S1. Clinical assessments. Table S2. Constructed haplotype with PHASE. Table S3. Correlation between scale-wise progression. Table S4. Correlation between raw PD progression and PCA. Table S5. PC variance. Table S6. Genotype distribution of 30 SNPs. Table S7. Diversity of gene-PD-progression correlation among diverse populations. Figure S1. Flowchart of the study. Figure S2. Chrome-wise pairwise LD. A: PARK16 haplotype; B: SNCA haplotype; C: chr6snps; D: chr12snps. Figure S3. Flowchart of SNP preprocessing. Figure S4. Heatmap of the contribution of raw scale progressions to PCs derived from three types of PCA. A: contribution of motor scales of PC from motor PCA; B: contribution of nonmotor scales to PC from nonmotor PCA; C: contribution of all scales to PC from composite PCA. Figure S5. PCA of progression rate and 30 SNPs. A. PCA of progression rate; B. PCA of 30 SNPs. [file 3481102.f1.docx]

Table S1. Clinic Assessments

| ID | Cohort | Clinical Assessments | SubScores | Function |
| --- | --- | --- | --- | --- |
| 1 | TW/PPMI | MDS-Unified Parkinson's Disease Rating Scale MDS-UPDRS | Part I | Spirit, Behavior and Emotion |
|  |  |  | Part II | patient-reported experiences of daily living |
|  |  |  | Part III | Clinician-assessed movement examination |
|  |  |  | Part IV |  |
| 2 | TW/PPMI | Hoehn-Yahr staging | / | Clinician-assessed rating of impairment and disability |
| 3 | TW/PPMI | The SCales for Outcomes in Parkinson's disease Autonomic disfunction SCOPA-AUT | Total score | Autonomic dysfunction |
| 4 | TW/PPMI | Epworth Sleepiness Scale ESS | Total score |  |
| 5 | TW/PPMI | The Hamilton Anxiety Rating Scale HAMA-HARMS/ State-Trait Anxiety Inventory for Adults STAI | Total score |  |
| 6 | TW/PPMI | Rapid Eye Movement Sleep Behavior Disorder Hongkong/Questionnaire RBD-HK/RBD-Q | Total score | Sleep Disorder |
| 7 | TW/PPMI | Montreal Cognitive Assessment MoCA | Total score |  |
| 8 | TW/PPMI | The Hamilton Rating Scale for Depression HAMD-17/ Geriatric Depression Scale GDS | Total score |  |

TableS2. Constructed Haplotype with PHASE

| Haplotype Group | SNP Set | Frequency |
| --- | --- | --- |
| *LRRK2* Haplotype (rs11564148-rs34778348) | TG | 0.617669 |
|  | AG | 0.37164 |
|  | AA | 0.009429 |
|  | TA | 0.001262 |
| *PARK16* Haplotype (rs823128-rs1572931-rs823144-rs947211) | ACAG | 0.74529 |
|  | ATCA | 0.133316 |
|  | GCCA | 0.060231 |
|  | ACCA | 0.047575 |
|  | ATCG | 0.004636 |
|  | ACCG | 0.003648 |
|  | GCAG | 0.001462 |
|  | ATAA | 0.001147 |
|  | GCCG | 0.00107 |
|  | ACAA | 0.000673 |
|  | GTCA | 0.000526 |
|  | ATAG | 0.000241 |
|  | GCAA | 0.000185 |
| *SNCA* Haplotype (rs356219-rs11931074-rs356221-rs356165-rs3822086-rs2736990) | AGTACA | 0.454786 |
|  | GGAGCG | 0.238178 |
|  | GTAGTG | 0.192366 |
|  | AGAACG | 0.099285 |
|  | AGTACG | 0.002018 |
|  | AGAGCG | 0.001786 |
|  | GTAATG | 0.001552 |
|  | GTAACA | 0.001343 |
|  | GTAGCA | 0.001116 |
|  | GTTACA | 0.001105 |
|  | AGTGCG | 0.001089 |
|  | AGAACA | 0.000936 |
|  | AGAGTG | 0.000679 |
|  | AGTATG | 6.59E-04 |
|  | ATAGTA | 6.57E-04 |
|  | ATAGCA | 6.41E-04 |
|  | GGAGCA | 5.73E-04 |
|  | GGAACA | 4.45E-04 |
|  | AGAATG | 4.34E-04 |
|  | GTAGTA | 2.78E-04 |
|  | ATAACA | 1.60E-05 |
|  | ATAGTG | 1.30E-05 |
|  | ATTGCA | 1.10E-05 |
|  | AGTGTG | 9.00E-06 |
|  | AGAGTA | 7.00E-06 |
|  | GGTGCG | 4.00E-06 |
|  | GTAACG | 4.00E-06 |
|  | GTTGTG | 0.000004 |

TableS3 Correlation between Scalewised progression

|  | Nonmotor Scales | | | | | | | Motor Scales | | | |
| --- | --- | --- | --- | --- | --- | --- | --- | --- | --- | --- | --- |
|  | SCOPA | ESS | MoCA | HAMD-17  or GDS | HAMA  or STAI | RBD-HK or RBD-Q | MDS-UPDRS I | MDS-UPDRS II | MDS-UPDRS III | MDS-UPDRS IV | Hoehn-Yahr staging |
| MDS-UPDRSI | 0.66 | 0.42 | -0.23 | 0.63 | 0.55 | 0.40 | 1.00 | 0.52 | 0.22 | 0.36 | 0.24 |
| MDS-UPDRSII | 0.51 | 0.33 | -0.31 | 0.40 | 0.30 | 0.29 | 0.52 | 1.00 | 0.48 | 0.28 | 0.38 |
| MDS-UPDRSIII | 0.23 | 0.10 | -0.30 | 0.17 | 0.12 | 0.11 | 0.22 | 0.48 | 1.00 | 0.14 | 0.65 |
| MDS-UPDRSIV | 0.23 | 0.16 | 0.01 | 0.23 | 0.15 | 0.15 | 0.36 | 0.28 | 0.14 | 1.00 | 0.24 |
| Hoehn-Yahr staging | 0.23 | 0.05 | -0.20 | 0.18 | 0.09 | 0.07 | 0.24 | 0.38 | 0.65 | 0.24 | 1.00 |
| SCOPA | 1.00 | 0.40 | -0.23 | 0.46 | 0.42 | 0.43 | 0.66 | 0.51 | 0.23 | 0.23 | 0.23 |
| ESS | 0.40 | 1.00 | -0.13 | 0.29 | 0.30 | 0.33 | 0.42 | 0.33 | 0.10 | 0.16 | 0.05 |
| MoCA | -0.23 | -0.13 | 1.00 | -0.21 | -0.18 | -0.11 | -0.23 | -0.31 | -0.30 | 0.01 | -0.20 |
| HAMD-17or GDS | 0.46 | 0.29 | -0.21 | 1.00 | 0.80 | 0.26 | 0.63 | 0.40 | 0.17 | 0.23 | 0.18 |
| HAMA or STAI | 0.42 | 0.30 | -0.18 | 0.80 | 1.00 | 0.29 | 0.55 | 0.30 | 0.12 | 0.15 | 0.09 |
| RBD-HK or  RBD-Q | 0.43 | 0.33 | -0.11 | 0.26 | 0.29 | 1.00 | 0.40 | 0.29 | 0.11 | 0.15 | 0.07 |

ESS, Epworth Sleepiness Scale Score ; GDS, Geriatric Depression Scale; HAMA, The Hamilton Anxiety Rating Scale; HAMD-17, The Hamilton Rating Scale for Depression; MDS-UPDRS, MDS-Unified Parkinson's Disease Rating Scale; MoCA, Montreal Cognitive Assessment; RBD-HK, Rapid Eye Movement Sleep Behavior Disorder Hongkong; RBD-Q, Rapid Eye Movement Sleep Behavior Disorder Questionnaire; SCOPA-AUT, Scales for Outcomes in Parkinson’s Disease – Autonomic Dysfunction; STAI, State-Trait Anxiety Inventory for Adults

Table S4 Correlation between raw PD progression and PCA

| Type | Scale | Dim.1 | Dim.2 | Dim.3 | Dim.4 | Dim.5 | Dim.6 | Dim.7 | Dim.8 | Dim.9 | Dim.10 | Dim.11 |
| --- | --- | --- | --- | --- | --- | --- | --- | --- | --- | --- | --- | --- |
| Composite PCA | MDS-UPDRSI | -0.83 | -0.19 | -0.08 | -0.06 | -0.06 | -0.03 | -0.19 | -0.14 | -0.04 | -0.44 | 0.07 |
|  | MDS-UPDRSII | -0.73 | 0.28 | -0.06 | 0.12 | -0.07 | 0.07 | -0.31 | 0.48 | -0.17 | 0.07 | 0.04 |
|  | MDS-UPDRSIII | -0.48 | 0.74 | 0.06 | 0.00 | 0.21 | 0.11 | 0.11 | 0.10 | 0.38 | -0.10 | -0.03 |
|  | MDS-UPDRSIV | -0.41 | 0.06 | -0.67 | -0.35 | -0.42 | -0.19 | 0.18 | 0.03 | 0.09 | 0.08 | 0.00 |
|  | Hoehn-Yahr staging | -0.45 | 0.72 | -0.09 | -0.17 | 0.21 | 0.06 | 0.12 | -0.28 | -0.31 | 0.07 | 0.03 |
|  | SCOPA | -0.76 | -0.12 | -0.08 | 0.21 | 0.05 | -0.08 | -0.41 | -0.31 | 0.16 | 0.24 | -0.02 |
|  | ESS | -0.54 | -0.25 | -0.16 | 0.43 | -0.16 | 0.59 | 0.26 | -0.05 | -0.02 | 0.02 | -0.02 |
|  | HAMA or STAI | -0.68 | -0.39 | 0.32 | -0.36 | 0.14 | 0.04 | 0.18 | 0.06 | 0.06 | 0.12 | 0.27 |
|  | RBD-HK or RBD-Q | -0.52 | -0.24 | -0.20 | 0.47 | 0.36 | -0.43 | 0.29 | 0.10 | -0.05 | -0.01 | -0.03 |
|  | MoCA | 0.39 | -0.29 | -0.58 | -0.28 | 0.51 | 0.26 | -0.15 | 0.08 | 0.02 | -0.01 | 0.00 |
|  | HAMD-17 or GDS | -0.75 | -0.31 | 0.27 | -0.41 | 0.06 | 0.03 | 0.06 | 0.05 | -0.05 | 0.02 | -0.31 |
| Nonmotor PCA | MDS-UPDRSI | 0.85 | -0.01 | -0.02 | 0.04 | 0.30 | 0.42 | -0.10 | \ | \ | \ | \ |
|  | SCOPA | 0.77 | -0.23 | 0.08 | 0.16 | 0.46 | -0.33 | 0.01 | \ | \ | \ | \ |
|  | ESS | 0.59 | -0.43 | 0.04 | -0.67 | -0.12 | -0.02 | 0.02 | \ | \ | \ | \ |
|  | HAMA or STAI | 0.78 | 0.41 | -0.26 | -0.03 | -0.25 | -0.15 | -0.27 | \ | \ | \ | \ |
|  | RBD-HK or RBD-Q | 0.57 | -0.56 | -0.02 | 0.44 | -0.41 | 0.04 | 0.03 | \ | \ | \ | \ |
|  | MoCA | -0.36 | -0.25 | -0.89 | -0.02 | 0.13 | 0.00 | 0.01 | \ | \ | \ | \ |
|  | HAMD-17 or GDS | 0.81 | 0.44 | -0.21 | -0.01 | -0.08 | 0.01 | 0.32 | \ | \ | \ | \ |
| Motor PCA | MDS-UPDRSII | -0.75 | -0.09 | 0.65 | 0.13 | \ | \ | \ | \ | \ | \ | \ |
|  | MDS-UPDRSIII | -0.84 | 0.34 | -0.09 | -0.41 | \ | \ | \ | \ | \ | \ | \ |
|  | MDS-UPDRSIV | -0.45 | -0.87 | -0.20 | -0.09 | \ | \ | \ | \ | \ | \ | \ |
|  | Hoehn-Yahr staging | -0.83 | 0.21 | -0.39 | 0.36 | \ | \ | \ | \ | \ | \ | \ |

ESS, Epworth Sleepiness Scale Score ; GDS, Geriatric Depression Scale; HAMA, The Hamilton Anxiety Rating Scale; HAMD-17, The Hamilton Rating Scale for Depression; MDS-UPDRS, MDS-Unified Parkinson's Disease Rating Scale; MoCA, Montreal Cognitive Assessment; PCA, principal components analysis; RBD-HK, Rapid Eye Movement Sleep Behavior Disorder Hongkong; RBD-Q, Rapid Eye Movement Sleep Behavior Disorder Questionnaire; SCOPA-AUT, Scales for Outcomes in Parkinson’s Disease – Autonomic Dysfunction; STAI, State-Trait Anxiety Inventory for Adults

TableS5. PC variance

| Type | PC | Eigenvalue | Variance Percentage | Cumulative Variance Percentage |
| --- | --- | --- | --- | --- |
| Composite PCA | Dim.1 | 4.14 | 37.61 | 37.61 |
|  | Dim.2 | 1.64 | 14.90 | 52.50 |
|  | Dim.3 | 1.05 | 9.51 | 62.01 |
|  | Dim.4 | 0.98 | 8.91 | 70.93 |
|  | Dim.5 | 0.72 | 6.52 | 77.45 |
|  | Dim.6 | 0.67 | 6.07 | 83.52 |
|  | Dim.7 | 0.57 | 5.17 | 88.69 |
|  | Dim.8 | 0.46 | 4.18 | 92.87 |
|  | Dim.9 | 0.31 | 2.83 | 95.70 |
|  | Dim.10 | 0.29 | 2.65 | 98.35 |
|  | Dim.11 | 0.18 | 1.65 | 100.00 |
| Motor PCA | Dim.1 | 2.15 | 53.69 | 53.69 |
|  | Dim.2 | 0.92 | 22.98 | 76.66 |
|  | Dim.3 | 0.61 | 15.28 | 91.95 |
|  | Dim.4 | 0.32 | 8.05 | 100.00 |
| Nonmotor PCA | Dim.1 | 3.37 | 48.18 | 48.18 |
|  | Dim.2 | 0.97 | 13.91 | 62.09 |
|  | Dim.3 | 0.91 | 13.03 | 75.12 |
|  | Dim.4 | 0.67 | 9.54 | 84.66 |
|  | Dim.5 | 0.57 | 8.21 | 92.88 |
|  | Dim.6 | 0.31 | 4.47 | 97.35 |
|  | Dim.7 | 0.19 | 2.65 | 100.00 |

Table S6. Genotype Distribution of 30 SNPs

| SNPs | Genotype | Alleles | TW (N=50) | | PPMI (N=413) | |
| --- | --- | --- | --- | --- | --- | --- |
|  |  |  | Genotype Freq | Major Allele Freq | Genotype Freq | Major Allele Freq |
| rs11564148 | T/T | T/A | 20 | 63 | 209 | 71.1 |
| rs11564148 | T/A | T/A | 23 | 63 | 169 | 71.1 |
| rs11564148 | A/A | T/A | 7 | 63 | 35 | 71.1 |
| rs11931074 | T/T | T/G | 23 | 65 | 3 | 88.9 |
| rs11931074 | G/T | T/G | 19 | 65 | 86 | 88.9 |
| rs11931074 | G/G | T/G | 8 | 65 | 324 | 88.9 |
| rs12456492 | A/A | A/G | 15 | 56 | 195 | 67.8 |
| rs12456492 | A/G | A/G | 26 | 56 | 170 | 67.8 |
| rs12456492 | G/G | A/G | 9 | 56 | 48 | 67.8 |
| rs12817488 | G/G | G/A | 17 | 53 | 116 | 52.5 |
| rs12817488 | G/A | G/A | 19 | 53 | 202 | 52.5 |
| rs12817488 | A/A | G/A | 14 | 53 | 95 | 52.5 |
| rs156429 | T/T | T/C | 28 | 74 | 148 | 60.7 |
| rs156429 | T/C | T/C | 18 | 74 | 205 | 60.7 |
| rs156429 | C/C | T/C | 4 | 74 | 60 | 60.7 |
| rs1572931 | C/C | C/T | 27 | 75 | 306 | 85.8 |
| rs1572931 | C/T | C/T | 21 | 75 | 97 | 85.8 |
| rs1572931 | T/T | C/T | 2 | 75 | 10 | 85.8 |
| rs1799836 | T/T | T/C | 38 | 82 | 181 | 52.5 |
| rs1799836 | T/C | T/C | 6 | 82 | 72 | 52.5 |
| rs1799836 | C/C | T/C | 6 | 82 | 160 | 52.5 |
| rs1801474 | T/T | T/C | 15 | 51 | 1 | 97.6 |
| rs1801474 | C/T | T/C | 21 | 51 | 18 | 97.6 |
| rs1801474 | C/C | T/C | 14 | 51 | 394 | 97.6 |
| rs1801582 | G/G | G/C | 40 | 90 | 8 | 85 |
| rs1801582 | C/G | G/C | 10 | 90 | 108 | 85 |
| rs1801582 | C/C | G/C | / | / | 297 | 85 |
| rs2301134 | G/G | G/A | 46 | 96 | 108 | 50.8 |
| rs2301134 | G/A | G/A | 4 | 96 | 204 | 50.8 |
|  | A/A | G/A | / | / | 101 | 50.8 |
| rs2435207 | G/G | G/A | 32 | 80 | 195 | 67.6 |
| rs2435207 | G/A | G/A | 16 | 80 | 168 | 67.6 |
| rs2435207 | A/A | G/A | 2 | 80 | 50 | 67.6 |
| rs2736990 | G/G | G/A | 21 | 66 | 122 | 54.8 |
| rs2736990 | G/A | G/A | 24 | 66 | 209 | 54.8 |
| rs2736990 | A/A | G/A | 5 | 66 | 82 | 54.8 |
| rs3129882 | G/G | G/A | 20 | 67 | 101 | 52.4 |
| rs3129882 | A/G | G/A | 27 | 67 | 191 | 52.4 |
| rs3129882 | A/A | G/A | 3 | 67 | 121 | 52.4 |
| rs33949390 | G/G | G/C | 47 | 97 | 412 | 99.9 |
| rs33949390 | G/C | G/C | 3 | 97 | 1 | 99.9 |
| rs34778348 | G/G | G/A | 43 | 93 | 412 | 99.9 |
| rs34778348 | G/A | G/A | 7 | 93 | 1 | 99.9 |
| rs356165 | G/G | G/A | 22 | 64 | 84 | 54 |
| rs356165 | A/G | G/A | 20 | 64 | 212 | 54 |
| rs356165 | A/A | G/A | 8 | 64 | 117 | 54 |
| rs356219 | G/G | G/A | 21 | 63 | 84 | 54.1 |
| rs356219 | A/G | G/A | 21 | 63 | 211 | 54.1 |
| rs356219 | A/A | G/A | 8 | 63 | 118 | 54.1 |
| rs356221 | A/A | A/T | 23 | 68 | 122 | 54.7 |
| rs356221 | A/T | A/T | 22 | 68 | 208 | 54.7 |
| rs356221 | T/T | A/T | 5 | 68 | 83 | 54.7 |
| rs3766606 | G/G | G/T | 42 | 92 | 304 | 85.1 |
| rs3766606 | G/T | G/T | 8 | 92 | 95 | 85.1 |
| rs3766606 | T/T | G/T | / | / | 14 | 85.1 |
| rs3822086 | T/T | T/C | 21 | 63 | 3 | 88.9 |
| rs3822086 | C/T | T/C | 21 | 63 | 86 | 88.9 |
| rs3822086 | C/C | T/C | 8 | 63 | 324 | 88.9 |
| rs4273468 | A/A | A/G | 12 | 54 | 11 | 82.4 |
| rs4273468 | G/A | A/G | 30 | 54 | 123 | 82.4 |
| rs4273468 | G/G | A/G | 8 | 54 | 279 | 82.4 |
| rs4375 | T/T | T/C | 22 | 68 | 148 | 60.2 |
| rs4375 | T/C | T/C | 24 | 68 | 201 | 60.2 |
| rs4375 | C/C | T/C | 4 | 68 | 64 | 60.2 |
| rs4680 | G/G | G/A | 25 | 73 | 118 | 50.2 |
| rs4680 | G/A | G/A | 23 | 73 | 179 | 50.2 |
| rs4680 | A/A | G/A | 2 | 73 | 116 | 50.2 |
| rs6245 | C/C | C/T | 43 | 93 | 384 | 96.2 |
| rs6245 | C/T | C/T | 7 | 93 | 27 | 96.2 |
| rs6245 | T/T | C/T | / | / | 2 | 96.2 |
| rs669 | T/T | T/C | 40 | 90 | 195 | 67.8 |
| rs669 | T/C | T/C | 10 | 90 | 170 | 67.8 |
| rs669 | C/C | T/C | / | / | 48 | 67.8 |
| rs7903491 | A/A | A/G | 16 | 51 | 76 | 57 |
| rs7903491 | G/A | A/G | 19 | 51 | 203 | 57 |
| rs7903491 | G/G | A/G | 15 | 51 | 134 | 57 |
| rs823128 | A/A | A/G | 37 | 87 | 380 | 95.8 |
| rs823128 | A/G | A/G | 13 | 87 | 31 | 95.8 |
| rs823128 | G/G | A/G | / | / | 2 | 95.8 |
| rs823144 | A/A | A/C | 17 | 62 | 228 | 74.5 |
| rs823144 | A/C | A/C | 28 | 62 | 159 | 74.5 |
| rs823144 | C/C | A/C | 5 | 62 | 26 | 74.5 |
| rs947211 | G/G | G/A | 18 | 63 | 230 | 74.8 |
| rs947211 | G/A | G/A | 27 | 63 | 158 | 74.8 |
| rs947211 | A/A | G/A | 5 | 63 | 25 | 74.8 |

TableS7 Diversity of Gene-PD-Progression Correlation among Diverse Population

| Scale | Gene/LDBlock | SNP | Progression Type | AllAsian | PPMI.Caucasians | PPMI.Asian | TW |
| --- | --- | --- | --- | --- | --- | --- | --- |
| HAMD-17 or GDS | *LRRK2* | rs11564148 | Raw Progression | 0.275268 | 0.156839 | 0.044095 | 0.142998 |
| MDS-UPDRS4 | *SYT4.RIT2* | rs12456492 | Raw Progression | 0.281936 | 0.004160 | 0.816987 | 0.603900 |
| SCOPA | *SYT4.RIT2* | rs12456492 | Raw Progression | 0.298313 | 0.012223 | 0.898340 | 0.112821 |
| MDS-UPDRS2 | *SYT4.RIT2* | rs12456492 | Raw Progression | 0.074894 | 0.000612 | 0.568532 | 0.128080 |
| NHY | *SYT4.RIT2* | rs12456492 | Raw Progression | 0.025192 | 0.017657 | 0.036910 | 0.039320 |
| RBD-HK or RBD-Q | *CCDC62* | rs12817488 | Raw Progression | 0.406707 | 0.026402 | 0.326574 | / |
| MDS-UPDRS4 | *GPNMB* | rs156429 | Raw Progression | 0.774945 | 0.806013 | 0.005578 | 0.737760 |
| MDS-UPDRS4 | *RAB29* | rs1572931 | Raw Progression | 0.018197 | 0.708071 | 0.711333 | 0.109740 |
| MDS-UPDRS1 | *RAB29* | rs1572931 | Raw Progression | 0.040629 | 0.835585 | 0.294152 | / |
| MDS-UPDRS3 | *RAB29* | rs1572931 | Raw Progression | 0.514152 | 0.451955 | 0.019394 | 0.414316 |
| MDS-UPDRS2 | *MAOB* | rs1799836 | Raw Progression | 0.039985 | 0.537489 | 0.967200 | 0.028219 |
| MoCA | *MAOB* | rs1799836 | Raw Progression | 0.000027 | 0.586966 | 0.019034 | 0.000188 |
| SCOPA | *MAOB* | rs1799836 | Raw Progression | 0.112395 | 0.632915 | 0.518086 | 0.012261 |
| HAMA or STAI | *PRKN* | rs1801474 | Raw Progression | 0.289508 | 0.029309 | 0.390033 | / |
| RBD-HK or RBD-Q | *PRKN* | rs1801582 | Raw Progression | 0.000005 | 0.825626 | / | / |
| MDS-UPDRS4 | *PRKN* | rs1801582 | Raw Progression | 0.230591 | 0.835783 | / | 0.007370 |
| ESS | *PRKN* | rs1801582 | Raw Progression | 0.032667 | 0.454215 | / | / |
| HAMA or STAI | *PRKN* | rs1801582 | Raw Progression | 0.043502 | 0.768436 | / | / |
| ESS | *MAPT* | rs2435207 | Raw Progression | 0.178574 | 0.122904 | 0.006426 | / |
| RBD-HK or RBD-Q | *HLA-DRA* | rs3129882 | Raw Progression | 0.031873 | 0.162319 | 0.499179 | / |
| HAMD-17 or GDS | *LRRK2_R1628P.H* | rs33949390 | Raw Progression | 0.155613 | 0.785944 | / | 0.042853 |
| MoCA | *LOC105377329* | rs356219 | Raw Progression | 0.453913 | 0.946691 | 0.027147 | 0.431851 |
| MDS-UPDRS2 | *PARK7* | rs3766606 | Raw Progression | 0.324264 | 0.327555 | 0.040861 | 0.688824 |
| MDS-UPDRS1 | *PARK7* | rs3766606 | Raw Progression | 0.547008 | 0.796261 | 0.025326 | / |
| MDS-UPDRS | *PARK7* | rs3766606 | Raw Progression | 0.053242 | 0.779316 | 0.043606 | 0.317990 |
| MoCA | *BST1* | rs4273468 | Raw Progression | 0.193595 | 0.748095 | 0.026499 | 0.701021 |
| RBD-HK OR RBD-Q | *PLA2G6* | rs4375 | Raw Progression | 0.003060 | 0.086186 | 0.109534 | / |
| SCOPA | *PLA2G6* | rs4375 | Raw Progression | 0.954045 | 0.893859 | 0.004091 | 0.217417 |
| HAMA or STAI | *COMT* | rs4680 | Raw Progression | 0.017532 | 0.486860 | 0.106105 | / |
| NHY | *COMT* | rs4680 | Raw Progression | 0.717883 | 0.288416 | 0.024138 | 0.757227 |
| MDS-UPDRS3 | *COMT* | rs4680 | Raw Progression | 0.045670 | 0.851282 | 0.746009 | 0.032217 |
| MDS-UPDRS2 | *PTHLH* | rs6245 | Raw Progression | 0.409386 | 0.034830 | / | 0.343606 |
| MDS-UPDRS1 | *NUCKS1* | rs823128 | Raw Progression | 0.002370 | 0.800463 | 0.425639 | / |
| SS_16_or_UPSIT | *SYT4.RIT2* | rs12456492 | Raw Progression | / | / | / | 0.034001 |
| SS_16_or_UPSIT | *LRRK2_G2385R* | rs34778348 | Raw Progression | / | / | / | 0.036444 |
| SS_16_or_UPSIT | *PLA2G6* | rs4375 | Raw Progression | / | / | / | 0.045571 |
| MDS-UPDRS1 | *PARK16haplotype* | PARK16 haplotype.ACCA | Raw Progression | 0.000761 | 0.245908 | / | / |
| MDS-UPDRS1 | *PARK16haplotype* | PARK16 haplotype.GCCA | Raw Progression | 0.008719 | 0.245906 | / | / |
| MDS-UPDRS1 | *PARK16haplotype* | PARK16 haplotype.GCAG | Raw Progression | 0.000007 | 0.245886 | / | / |
| MDS-UPDRS1 | *PARK16haplotype* | PARK16 haplotype.GTCA | Raw Progression | 0.000889 | 0.245839 | / | / |
| MDS-UPDRS4 | *PARK16haplotype* | PARK16 haplotype.ACCA | Raw Progression | 0.010350 | 0.324145 | / | 0.347905 |
| MDS-UPDRS4 | *PARK16haplotype* | PARK16 haplotype.GCAG | Raw Progression | 0.003006 | 0.324293 | / | / |
| HAMD-17 or GDS | *SNCAhaplotype* | SNCA haplotype.GGAGCG | Raw Progression | 0.046407 | 0.501431 | / | / |
| HAMA or STAI | *SNCAhaplotype* | SNCA haplotype.GGAGCG | Raw Progression | 0.000306 | 0.855042 | / | / |
| RBD-HK OR RBD-Q | *SNCAhaplotype* | SNCA haplotype.GGAGCG | Raw Progression | 0.031783 | 0.928812 | / | / |
| NHY | *LRRK2haplotype* | LRRK2 haplotype.TG | Raw Progression | 0.027726 | 0.908681 | 0.145644 | 0.045844 |
| NHY | *LRRK2haplotype* | LRRK2 haplotype.AG | Raw Progression | 0.026722 | 0.926898 | / | 0.039827 |
| NHY | *LRRK2haplotype* | LRRK2 haplotype.AA | Raw Progression | 0.027801 | / | / | 0.043771 |
| HAMD-17 or GDS | *LRRK2haplotype* | LRRK2 haplotype.TG | Raw Progression | 0.659358 | 0.631078 | 0.044095 | 0.420946 |
| PC1 | *SYT4.RIT2* | rs12456492 | Motor PCA | 0.106243 | 0.000713 | 0.719826 | 0.197847 |
| PC1 | *MAOB* | rs1799836 | Composite PCA | 0.004163 | 0.705583 | 0.909089 | 0.001492 |
| PC1 | *MAOB* | rs1799836 | Motor PCA | 0.048769 | 0.484786 | 0.683104 | 0.042569 |
| PC1 | *MAOB* | rs1799836 | Nonmotor PCA | 0.004988 | 0.905945 | 0.996437 | 0.000077 |
| PC1 | *PRKN* | rs1801582 | Nonmotor PCA | 0.030113 | 0.792805 | / | 0.932809 |
| PC1 | *PLA2G6* | rs4375 | Motor PCA | 0.489286 | 0.164956 | 0.034641 | 0.858405 |
| PC1 | *NUCKS1* | rs823128 | Nonmotor PCA | 0.028573 | 0.583516 | 0.317928 | 0.170322 |
| PC2 | *LRRK2* | rs11564148 | Nonmotor PCA | 0.027308 | 0.184134 | 0.230025 | 0.138473 |
| PC2 | *SYT4.RIT2* | rs12456492 | Motor PCA | 0.918252 | 0.027401 | 0.786553 | 0.984546 |
| PC2 | *SYT4.RIT2* | rs12456492 | Motor PCA | 0.918252 | 0.027401 | 0.786553 | 0.984546 |
| PC2 | *MAOB* | rs1799836 | Nonmotor PCA | 0.000902 | 0.395333 | 0.605340 | 0.000069 |
| PC2 | *MAOB* | rs1799836 | Nonmotor PCA | 0.000902 | 0.395333 | 0.605340 | 0.000069 |
| PC2 | *PRKN* | rs1801582 | Motor PCA | 0.569879 | 0.290522 | / | 0.011221 |
| PC2 | *LRRK2_R1628P.H* | rs33949390 | Motor PCA | 0.069575 | 0.492014 | / | 0.048270 |
| PC2 | *LOC105377329* | rs356219 | Motor PCA | 0.589478 | 0.020171 | 0.950081 | 0.708774 |
| PC3 | *SYT4.RIT2* | rs12456492 | Composite PCA | 0.271313 | 0.000645 | 0.791832 | 0.166527 |
| PC3 | *MAOB* | rs1799836 | Nonmotor PCA | 0.000048 | 0.881860 | 0.406963 | 0.000008 |
| PC3 | *MAOB* | rs1799836 | Motor PCA | 0.094259 | 0.549679 | 0.927753 | 0.045572 |
| PC3 | *MAOB* | rs1799836 | Nonmotor PCA | 0.000048 | 0.881860 | 0.406963 | 0.000008 |
| PC3 | *MAPT* | rs2435207 | Motor PCA | 0.072444 | 0.015289 | 0.186490 | 0.266066 |
| PC3 | *PARK7* | rs3766606 | Motor PCA | 0.203298 | 0.055430 | 0.005345 | 0.408514 |
| PC3 | *COMT* | rs4680 | Nonmotor PCA | 0.957431 | 0.875719 | 0.035536 | 0.761144 |
| PC1 | *SNCAhaplotype* | SNCA haplotype.AGTGCA | Motor PCA | / | 0.001150 | / | / |
| PC1 | *LRRK2haplotype* | LRRK2 haplotype.AG | Composite PCA | 0.048415 | 0.254200 | / | 0.056940 |
| PC2 | *PARK16haplotype* | PARK16 haplotype.ACAG | Nonmotor PCA | 0.403869 | 0.025029 | / | 0.170447 |
| PC2 | *PARK16haplotype* | PARK16 haplotype.ACCA | Nonmotor PCA | 0.808736 | 0.025026 | / | 0.896103 |
| PC2 | *PARK16haplotype* | PARK16 haplotype.ACCG | Nonmotor PCA | 0.525479 | 0.025044 | / | 0.314628 |
| PC2 | *PARK16haplotype* | PARK16 haplotype.ATCA | Nonmotor PCA | 0.348914 | 0.025029 | / | 0.170191 |
| PC2 | *PARK16haplotype* | PARK16 haplotype.ATCG | Nonmotor PCA | 0.604873 | 0.025021 | / | 0.388113 |
| PC2 | *PARK16haplotype* | PARK16 haplotype.GCAG | Nonmotor PCA | 0.344536 | 0.025022 | / | / |
| PC2 | *PARK16haplotype* | PARK16 haplotype.GCCA | Nonmotor PCA | 0.249558 | 0.025030 | / | 0.683524 |
| PC2 | *PARK16haplotype* | PARK16 haplotype.GTCA | Nonmotor PCA | 0.466417 | 0.025009 | / | / |
| PC2 | *SNCAhaplotype* | SNCA haplotype.AGTGCA | Composite PCA | / | 0.000614 | / | / |
| PC2 | *SNCAhaplotype* | SNCA haplotype.GGAGCG | Nonmotor PCA | 0.009090 | 0.974666 | / | / |
| PC3 | *SNCAhaplotype* | SNCA haplotype.AGTGCA | Motor PCA | / | 0.000095 | / | / |

ESS, Epworth Sleepiness Scale Score ; GDS, Geriatric Depression Scale; HAMA, The Hamilton Anxiety Rating Scale; HAMD-17, The Hamilton Rating Scale for Depression; MDS-UPDRS, MDS-Unified Parkinson's Disease Rating Scale; MoCA, Montreal Cognitive Assessment; PC, principal components; RBD-HK, Rapid Eye Movement Sleep Behavior Disorder Hongkong; RBD-Q, Rapid Eye Movement Sleep Behavior Disorder Questionnaire; SCOPA-AUT, Scales for Outcomes in Parkinson’s Disease – Autonomic Dysfunction; STAI, State-Trait Anxiety Inventory for Adults


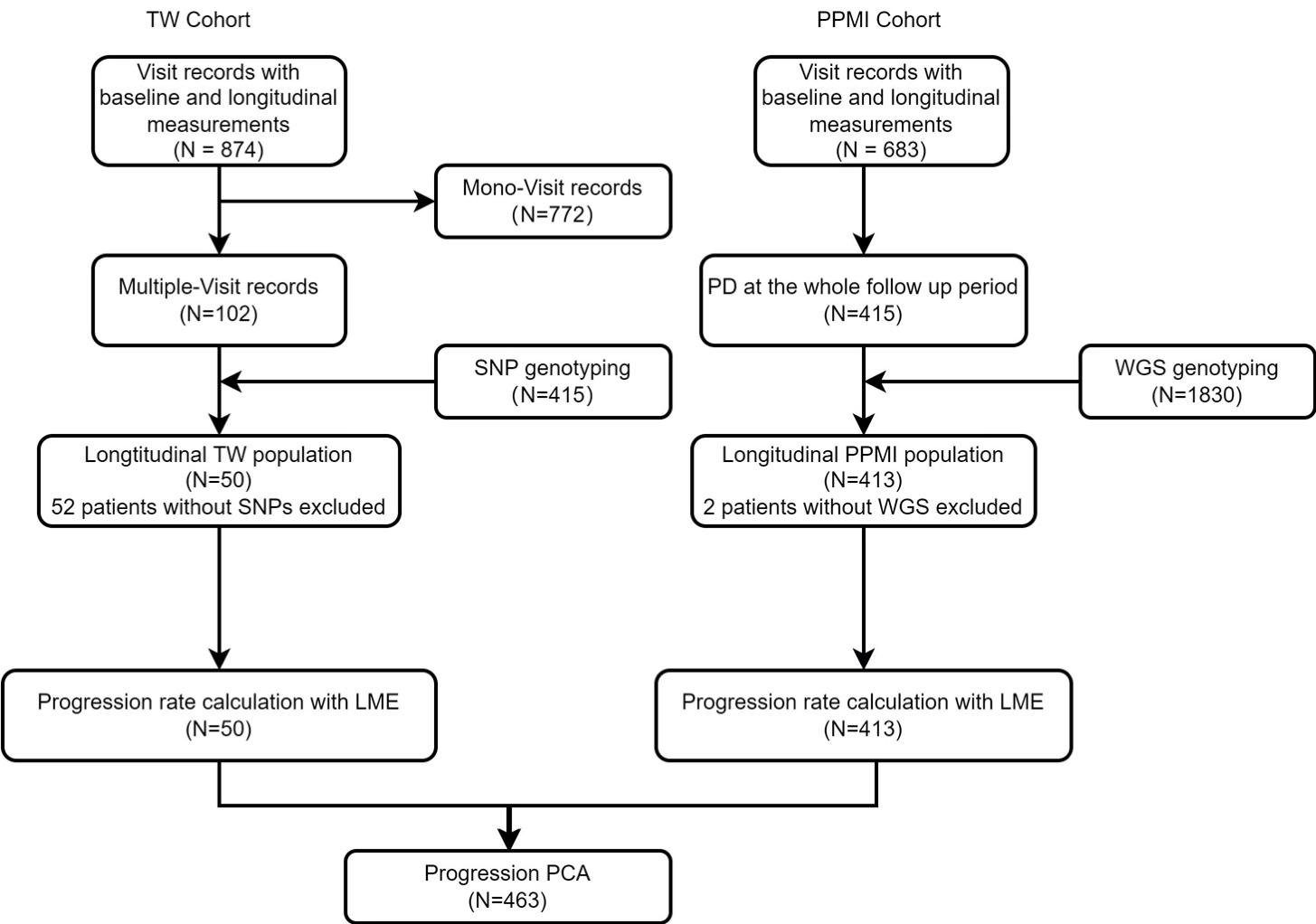


FigureS1. Flowchart of the Study.

In final, 463 PD patients were included for analyzing the genotype and phenotype correlation. All the genetic information requested from PPMI Cohort Genetic Database (N=1830), together with TW Cohort (N=415) were used to call haplotypes.


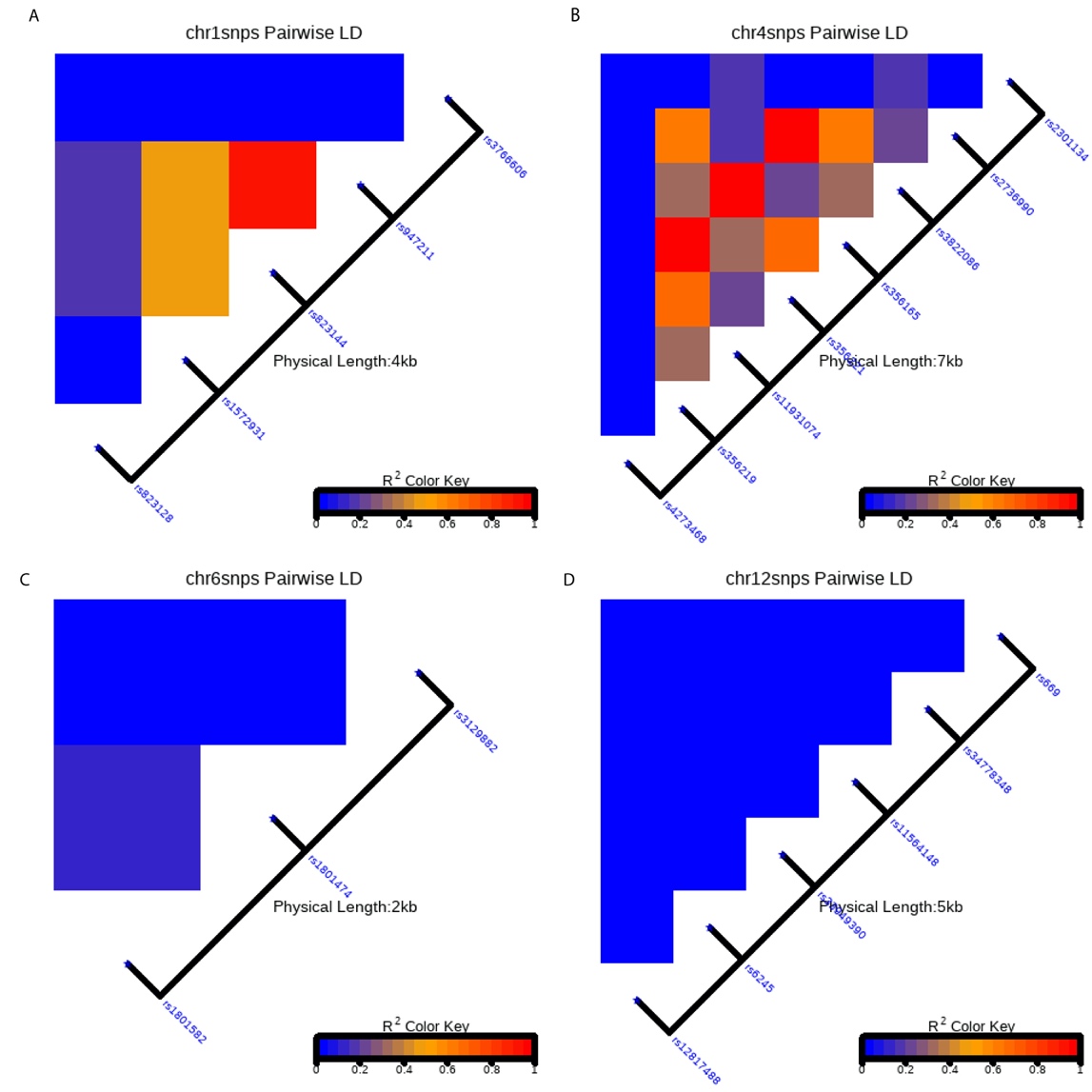


FigureS2.Chrome wised pairwise LD. A: *PARK16* haplotype; B: *SNCA* haplotype; C: chr6snps; D: chr12snps.


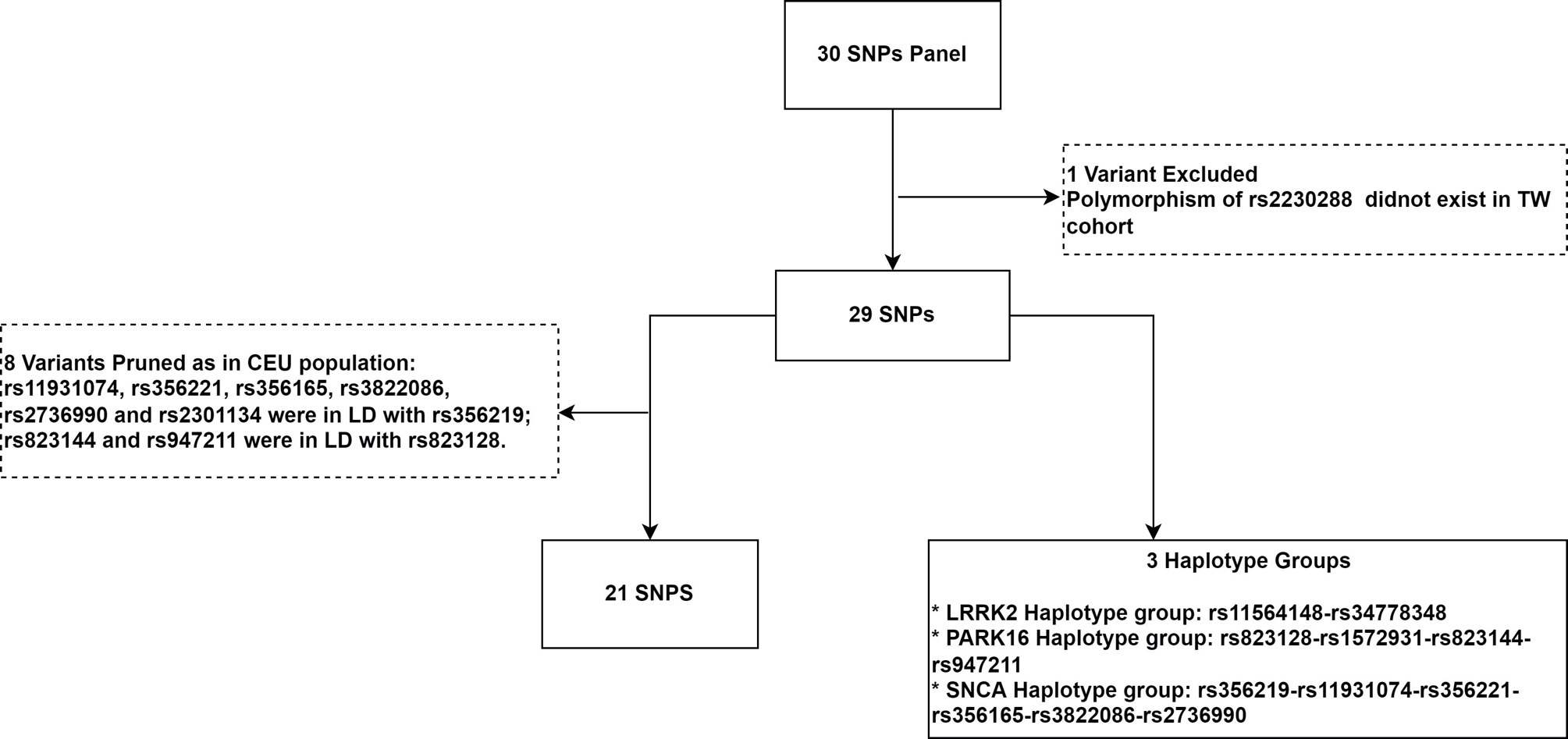


FigureS3. Flowchart of SNP preprocessing.


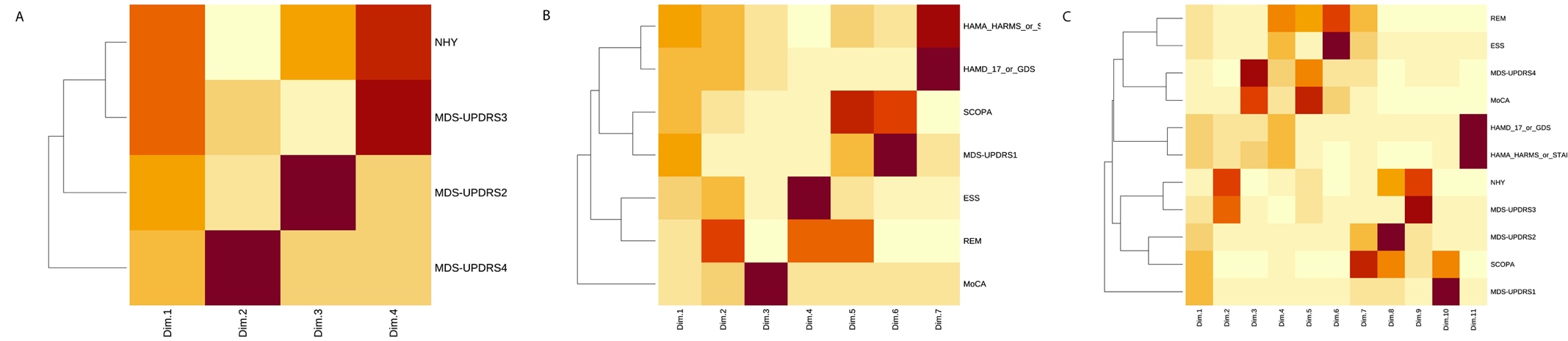


FigureS4. Heatmap of contribution of raw scale progressions to PCs derived from three types of PCA. A: contribution of motor scales to PC from motor PCA; B: contribution of nonmotor scales to PC from nonmotor PCA; C: contribution of all scales to PC from composite PCA.


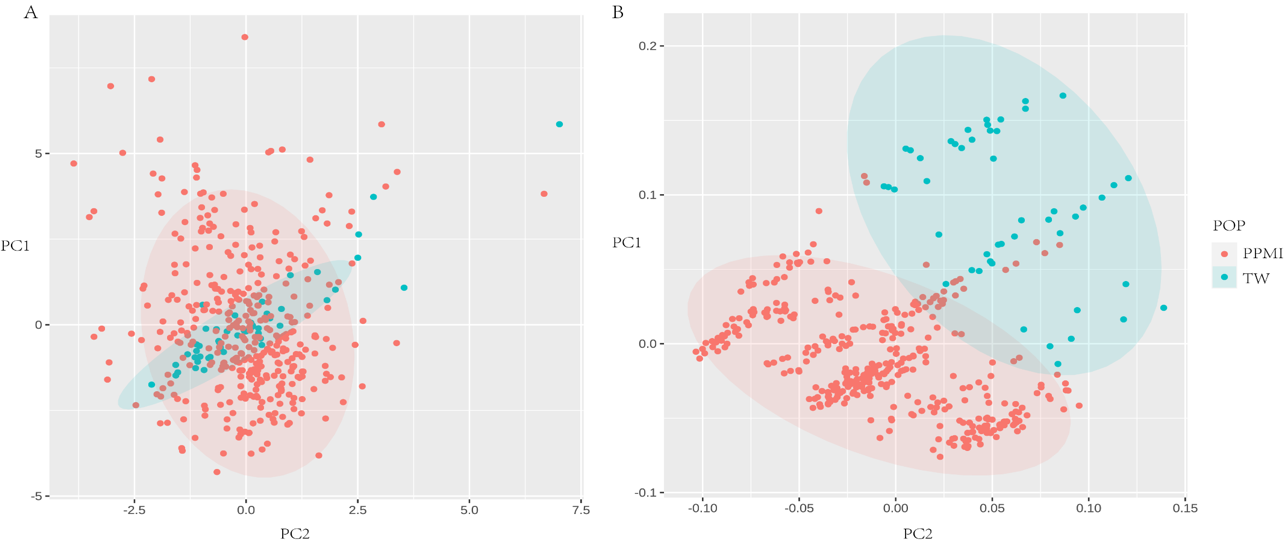


FigureS5. PCA of Progression Rate and 30 SNPs. A. PCA of Progression Rate; B. PCA of 30 SNPs.
